# The antecedents and consequences of autonomous and controlled motivation: Domain specificity and motivational sequence at the situational level

**DOI:** 10.3389/fpsyg.2022.987582

**Published:** 2022-09-29

**Authors:** Delphine Paumier, Julien Chanal

**Affiliations:** ^1^Faculty of Psychology and Educational Sciences, University of Geneva, Geneva, Switzerland; ^2^Faculty of Psychology, Distance Learning University, Brig, Switzerland

**Keywords:** self-determination theory, school-subject-specificity hypothesis, autonomy-supportive climate, academic emotions, achievement

## Abstract

The aim of this study was to investigate the level of specificity of the different regulation types described by Self-Determination Theory, and to evaluate its impact on the links with its antecedents and consequences, in an academic context. In line with the school-subject-specificity hypothesis, we postulated that autonomous motivation types (AM types) would be more specific to the situational level than controlled motivation types (CM types). Moreover, we hypothesized that AM types would be, at this level, more strongly associated with its antecedents and consequences than CM types. Three hundred fourteen university students were asked to complete online questionnaires assessing their motivation, motivational antecedents (i.e., autonomy-supportive climate and self-concept) and consequences (i.e., emotions and grades) in various courses. As expected, results from structural equation modeling confirmed that AM types were more specific to the situational level than CM types. Moreover, a higher number of significant correlations were found between motivation and its antecedents and consequences in the corresponding course for AM than for CM types. Educational implications and directions for future research are discussed.

## Introduction

Self-Determination Theory (SDT; Deci and Ryan, 1985; Ryan and Deci, 2017) provides a framework that enables to understand the mechanisms favoring optimal functioning. Different regulation types have been described, varying in their level of self-determination, from the most autonomous to the most controlled. Moreover, SDT describes how social contexts influence motivation. More specifically, social environments that facilitate the satisfaction of basic psychological needs promote the most autonomous motivation types (Deci and Ryan, 2000). In contrast, social contexts which thwart satisfaction of these needs yield the most controlled motivation types (Deci and Ryan, 2000). Autonomous motivation types (AM types) have been shown to be associated with greater creativity, enhanced psychological well-being, more persistence and higher performance in activities (Deci and Ryan, 2008) whereas controlled motivation types (CM types) have been associated with lower well-being, poorer mental health and lower performances in activities (see Deci and Ryan, 2000, for a review).

Recently, in the academic domain, the school-subject-specificity hypothesis (Chanal and Guay, 2015) was developed to explain why the differentiation between school subjects was different according to the types of motivation. Specifically, AM and CM types were demonstrated to be not equally specific to the school subject (i.e., the situational level) in which they were assessed. Using a multiple school subjects and multiple level of hierarchy design (i.e., situational and contextual), AM types were found to be more differentiated across school subjects than CM types because AM types were more specific to the situational level than CM types. These results could have serious implications on the relations between antecedents and consequences of AM and CM types in the SDT framework. Indeed, this would imply that antecedents and consequences should be more related to AM than to CM types at the situational level. However, results in studies aimed at exploring relations between antecedents and consequences with AM and CM types at the situational level had never examined a difference in the specificity level of the motivation types. Because most of the SDT research had focused on studies in situational level or in contextual level separately, or combined AM and CM types to operationalize motivation at the situational level (i.e., by using an index) without considering this specificity difference, questions about the consequences of this result on the motivational classsical sequence depicted in SDT are still unanswered.

The aim of this study was to investigate antecedents and consequences of AM and CM types in light of the school-subject-specificity hypothesis. More precisely, we wanted to examine whether the nature and the strength of relations between antecedents and consequences of motivation could depend on the specificity of the motivation types. In this research, we thus considered antecedents (i.e., autonomy-supportive climate, self-concept) and consequences (i.e., emotions and grades) of students’ motivation types at the situational level (i.e., toward various university courses) controlling for the level of specificity of the different measures by considering the contextual level (i.e., motivation for studying psychology).

### Self-determination theory

Contrary to other motivational theories that have treated motivation as a unitary concept varying only in quantity (e.g., Bandura, 1997), Self-Determination Theory considers motivation as a multidimensional concept that also varies in terms of quality. Indeed, SDT recognizes that there are three types of motivation: Intrinsic motivation, extrinsic motivation and amotivation (Ryan and Deci, 2017). Intrinsic motivation refers to the act of doing an activity for the pleasure and for its inherent satisfaction (Ryan and Deci, 2000a). On the contrary, extrinsic motivation is defined as doing of an activity to attain some separable consequences (Ryan and Deci, 2017). Deci and Ryan (1985) suggest the existence of different types of extrinsic motivation varying in their level of self-determination. Four extrinsic motivations are considered from low to high level of self-determination: External regulation, introjected regulation, identified regulation, and integrated regulation. External regulation involves doing a behavior to satisfy an external demand or reward contingency (Ryan and Deci, 2000b). Introjected regulation is the second type of extrinsic motivation and occurs when behaviors are performed in response to internal pressures, to avoid guilt or anxiety or to attain pride or ego-enhancements (Ryan and Deci, 2000a). Recently, Assor et al. (2009) distinguished between two types of introjected regulation: Introjected approach (or positive introjected) and introjected avoidance (or negative introjected). Introjected approach refers to the act of doing an activity to attain feelings of high self-worth or pride, whereas introjected avoidance refers to the act of doing an activity to avoid feelings of low self-worth, shame or guilt (Assor et al., 2009). According to these authors, introjected approach would be more self-determined than introjected avoidance. A more self-determined form of extrinsic motivation is identified regulation. Identified regulation occurs when people have identified with the personal importance of a behavior and have accepted it as their own (Ryan and Deci, 2017). The behavior is freely chosen but it is performed for instrumental purposes. The most self-determined form of extrinsic motivation is integrated regulation. Integrated regulation occurs when identifications have been evaluated and brought into congruence with individual’s values and needs (Ryan and Deci, 2002). As explained by Sheldon et al. (2017), there is a consensus that integrated motivation is problematic to measure. Other than the classical intrinsic/extrinsic one, another distinction occurs in the SDT framework relative to the level of internalization of motivation types. Autonomous motivation types refer to behaviors performed voluntarily and by choice (Vansteenkiste et al., 2006), and comprises intrinsic motivation as well as integrated and identified regulations. In contrast, controlled motivation types refer to behaviors constrained by internal and external pressures (Assor et al., 2009), and comprises introjected and external regulations. Finally, amotivation refers to a lack of intentionality or a lack of motivation (Deci and Ryan, 2008).

Given the multidimensional nature of motivation in the SDT, the level of individuals’ motivation has been operationalized in different ways. Some research (e.g., Chanal and Guay, 2015) has considered the motivation types proposed by the SDT separately (i.e., using the subscales as separate variables). Some others (e.g., Black and Deci, 2000) have created composite scores based on measurements of these motivation types (e.g., the Relative Autonomy Index (RAI) obtained by weighting the scores obtained on the different regulations according to their degree of self-determination^1^). The use of the RAI is justified by the fact that it reflects the continuum structure of motivation. Lastly, some others (e.g., De Naeghel et al., 2012) created a composite score based on autonomous motivation (AM) (e.g., by calculating the average obtained for intrinsic, integrated and identified regulations) and controlled motivation (CM) (e.g., by calculating the average obtained for introjected and external regulations). The use of AM and CM scores is supported by evidence for a second-order factor structure (e.g., Gagné et al., 2015).

### Hierarchical model of intrinsic and extrinsic motivation

Within the SDT framework, the Hierarchical Model of Intrinsic and Extrinsic Motivation (HMIEM; Vallerand, 1997) was developed with the aim to propose an integrative model able to describe the mechanisms of antecedents and consequences of motivation at different hierarchical levels. First, the HMIEM takes into consideration the different forms of motivation described by SDT and highlights the motivational sequence between antecedents and consequences of motivation. Second, this model considers that these types of motivation exist at three different hierarchical levels. These levels are described as: The *global*, the *contextual* and the *situational*. The global motivation level is defined as a broad disposition to engage in an activity according to an intrinsic, extrinsic or amotivated way (Vallerand, 2000). It refers to individual differences in motivation and can be seen as a characteristic of personality (Vallerand, 1997). The contextual motivation level refers to “motivational orientations (…) that are specific to various contexts such as education, leisure, and interpersonal relationships” (Vallerand, 2000, p. 313). The contextual motivation may vary drastically from one context to another and is more subject to variations than the global motivation (Vallerand, 1997). The situational motivation level refers to the motivation when people are currently engaging in an activity and corresponds to the here and now of motivation (Vallerand, 1997). According to the model, the motivational sequence also exists at these three hierarchical levels. More precisely, antecedents at a particular hierarchical level are described as influencing motivation at the corresponding level, and motivation at a particular hierarchical level as inducing consequences at that corresponding level. Motivational antecedents refer to social factors, human or nonhuman, found in our social environment (Vallerand, 1997). Note that motivation at a given level of the hierarchy is also influenced by motivation at the higher level (e.g., motivation at the situational level influences motivation at the contextual level) (Vallerand, 2000).

### Antecedents of autonomous and controlled motivation

As mentioned above, social factors are considered as antecedents of motivation. More specifically, according to Vallerand (1997) and Deci and Ryan (2008), social factors influence individual’s motivation through their impact on the satisfaction of basic psychological needs (i.e., need for autonomy, competence, and relatedness). Indeed, social factors that satisfy people’s basic psychological needs, lead to the most autonomous motivation types (Ryan and Deci, 2000b). In contrast, social factors that thwart satisfaction of these needs yield the least autonomous motivation types (Ryan and Deci, 2000b). Many studies in different contexts (e.g., sport, health, work, education) have therefore focused on the environmental conditions that support people’s psychological needs. In the academic context, one of the most studied social factors influencing students’ motivation is the motivational climate introduced by the teacher in the classroom and especially the teaching style he or she uses (Vallerand and Miquelon, 2016). Teachers’ motivational climate is postulated to influence students’ motivation by satisfying or thwarting their need for autonomy (Vallerand, 1997; Ryan and Deci, 2017). The need for competence is another psychological need that has often been studied in the academic context. The satisfaction or frustration of this need in students is assumed to influence their academic motivation (Vallerand, 1997; Ryan and Deci, 2017). In many studies, the feelings or perceptions of competence have been studied and operationalized as a more general construct namely the self-concept. In the academic context, these two antecedents (i.e., motivational climate and self-concept) have been studied at the contextual (i.e., toward school in general) and situational levels (i.e., toward a specific school subject or course). These antecedents are discussed in the following sections.

#### Teachers’ motivational climate

Teachers’ motivational climate refers to the interpersonal style that teachers adopt in the classroom with their students. These motivational interpersonal styles of teachers range along a continuum that goes from a style conceptualized as controlling to a style conceptualized as autonomy-supportive (Reeve and Jang, 2006; Reeve, 2015). Autonomy-supportive teachers offer choices to students, acknowledge their affects and feelings, and explain the use, value, and importance of school activities (Reeve, 2006). In contrast, controlling teachers provide extrinsic incentives, emphasize external evaluations, use pressuring communications and establish external goals (Reeve and Jang, 2006; Reeve, 2015).

Studies considering a combination of all motivation types into a single composite score consistently showed that the more students felt their autonomy supported by their teacher, the higher their RAI toward studies at the contextual level (Soenens and Vansteenkiste, 2005; Amoura et al., 2015; Orsini et al., 2017) and at the situational level (Black and Deci, 2000; Filak and Sheldon, 2008). When research considered AM and CM, results showed that autonomy-supportive climate was consistently positively associated with AM at the contextual level (Vansteenkiste et al., 2012; Litalien and Guay, 2015; Orsini et al., 2017) and at the situation level (Haerens et al., 2015; Behzadnia et al., 2018). However, results were less consistent for CM at both levels, showing negative (Litalien and Guay, 2015) or positive relation (Orsini et al., 2017) with autonomy-supportive climate, or no significant association between these constructs (Vansteenkiste et al., 2012). Research considering each motivation type separately were only find at the situational level. The results showed consistent positive associations between autonomy-supportive climate and intrinsic and identified regulations (Guay et al., 2013; Sparks et al., 2016; Behzadnia et al., 2018; Vasconcellos et al., 2020) while the relations between motivational climate and introjected and external regulations were more mixed. Introjected regulation was unrelated to autonomy-supportive climate in some studies (Behzadnia et al., 2018; Vasconcellos et al., 2020), while in other studies positive associations were found (Guay et al., 2013; Sparks et al., 2016). Concerning external regulation, results of studies showed that this regulation was negatively associated with autonomy-supportive climate in some studies (Sparks et al., 2016; Behzadnia et al., 2018) and unrelated in other study (Vasconcellos et al., 2020). Finally, a meta-analysis (Bureau et al., 2022), carried out at the two levels of hierarchy, showed that autonomy support from teacher was positively related to intrinsic motivation, identified, and introjected regulations, and negatively to external regulation.

#### Student’s self-concept

Self-concept can broadly be described as individuals’ perceptions of themselves, formed through experience and interpretation of their environment (Shavelson et al., 1976). These perceptions comprise feelings of self-confidence, self-worth, self-acceptance, competence, and ability (Marsh et al., 2017). In the academic context, the self-concept mainly refers to the students’ perceptions of their competences in school or in their studies in general (i.e., contextual level) or in a specific school subject or course (i.e., situational level).

Studies using a single composite score showed that academic self-concept was positively associated with RAI at the contextual level (Fortier et al., 1995; Vallerand et al., 1997; Guay et al., 2010b) and at the situational level (Timo et al., 2016). Considering AM and CM separately, results were more mixed whether they were at the contextual or situation level (De Naeghel et al., 2012; Valenzuela et al., 2018). Few studies (Guay et al., 2010a; Chanal and Guay, 2015) examined the links between each motivation type separately and self-concept at school level (Valenzuela et al., 2018) or in various school subjects simultaneously (Guay et al., 2010a; Chanal and Guay, 2015). Results showed consistent evidence that self-concepts were positively associated with intrinsic motivation and identified regulation in a corresponding school subject, whereas relations between self-concepts and introjected and external regulations in a corresponding subject were more mixed showing either no relations or positive ones.

### Consequences of autonomous and controlled motivation

Finally, motivation produces important consequences that can be affective (e.g., interest, emotions, satisfaction), cognitive (e.g., concentration, attention, learning) and behavioral (e.g., persistence in the task, performance) (Vallerand, 1997). More importantly, a key idea of SDT and HMIEM is that the different regulation types lead to different consequences. Indeed, they postulate that the more self-determined the motivation is, the more positive the consequences are. In the following sections, we will focus on the studies concerning the consequences in terms of academic achievement and emotions and will present them according to the hierarchical level considered and the operationalization of motivation used.

#### Student’s achievement

All studies using a single composite score at the contextual or situational level demonstrated that the higher the RAI, the higher achievement in general (Grolnick et al., 1991; Fortier et al., 1995; Guay and Vallerand, 1997; Black and Deci, 2000; Ratelle et al., 2005). Research considering AM and CM separately confirmed the positive influence of AM at the contextual (Brunet et al., 2015; Litalien et al., 2015) and situational level (De Naeghel et al., 2012; Jeno et al., 2018; Botnaru et al., 2021) and demonstrated the negative impact of CM on academic achievement except for one study at the situational level (Jeno et al., 2018). However, other studies found no significant relations between achievement and CM (Kusurkar et al., 2013). For studies considering each regulation type separately, results confirmed that academic achievement was positively associated with intrinsic and identified regulations at both levels (Noels et al., 2001; Taylor et al., 2014; Litalien et al., 2015; Leroy and Bressoux, 2016; Lohbeck, 2018; Orsini et al., 2019; Howard et al., 2021, study 1). Concerning CM types, results were more mixed and showed that achievement was negatively associated with introjected and external regulations in some studies (Taylor et al., 2014; Litalien et al., 2015; Leroy and Bressoux, 2016; Lohbeck, 2018, study 1), while in others (Noels et al., 1999; McEown et al., 2014; Orsini et al., 2019; Howard et al., 2021) no significant relations were found for these two regulations.

#### Student’s academic emotions

All studies using a single composite score at the contextual or situational level demonstrated that the higher the students’ RAI, the more positive emotions they experienced (Miserandino, 1996; Black and Deci, 2000; Levesque et al., 2004). Research considering AM and CM separately confirmed the positive influence of AM and demonstrated the negative impact of CM on different affects (Brunet et al., 2015; Litalien et al., 2015) at the contextual level. For studies considering each motivation type separately, research showed that autonomous motivation types were related to the most positive consequences and that external regulation was related to negative outcomes at both level (Noels et al., 1999; Litalien et al., 2015; Howard et al., 2021). Results are mixed for introjected regulations. Some studies showed that introjected regulation was negatively related to positive affective outcomes, and positively related to negative affective outcomes (Noels et al., 1999; Litalien et al., 2015; Howard et al., 2021) but some others found introjected regulation to be positively related to pleasure in school activities (Ryan and Connell, 1989; Howard et al., 2021) and to positive emotions (Vallerand et al., 1989; Bailey and Phillips, 2016; Howard et al., 2021).

In sum, it seems that the links between the different types of motivation and their antecedents and consequences seem to be dependent of the operationalization of the motivation used. Indeed, the results of studies using a single composite score (i.e., RAI) are consistent with each other and with the assumptions of SDT. On the other hand, when the types of motivations are operationalized in two composite scores (i.e., AM and CM) or when the types of motivation are considered separately, the results appear more nuanced and sometimes contradict theoretical assumptions but also each other. Second, in most of the studies presented, the authors assessed motivation, antecedents, and consequences at only one hierarchical level or in one school subject. Indeed, few studies have examined the links between motivation, its antecedents, and consequences at different hierarchical levels or toward several school subjects simultaneously. However, these links also seem to depend on the hierarchical level that is considered. Tables 1, 2 detail the results of studies presented in this article on the relationships between motivation and its antecedents (Table 1) and its consequences (Table 2). It should be noted that given the large number of publications available, the list of studies presented is not completely exhaustive but provides a good overview of the results concerning the links between motivation and its antecedents and consequences in the educational context.

**Table 1:** Synthesis of the links observed in the studies between motivation and its antecedents according to the hierarchical level considered and the operationalization of the motivation used.

	Antecedents			
	Motivational climate		Self-concept	
	Contextual	Situational	Contextual	Situational
RAI	*Positive* (Soenens and Vansteenkiste, 2005; Amoura et al., 2015; Orsini et al., 2017)	*Positive* (Black and Deci, 2000; Filak and Sheldon, 2008)	*Positive* (Fortier et al., 1995; Vallerand et al., 1997; Guay et al., 2010a)	*Positive* (Timo et al., 2016)
AM and CM				
AM	*Positive* (Vansteenkiste et al., 2012; Litalien and Guay, 2015; Orsini et al., 2017)	*Positive* (Haerens et al., 2015; Behzadnia et al., 2018)	*NS* (Valenzuela et al., 2018)	*Positive* (De Naeghel et al., 2012)
CM	*Positive* (Orsini et al., 2017) *Negative* (Litalien and Guay, 2015) *NS* (Vansteenkiste et al., 2012)	*NS* (Haerens et al., 2015; Behzadnia et al., 2018)	*Positive* (Valenzuela et al., 2018)	*Negative* (De Naeghel et al., 2012) *Positive* (2/3 SS) (Guay et al., 2010a) *NS* (1/3 SS) (Guay et al., 2010a)
Regulations separately				
Intrinsic		*Positive* (Guay et al., 2013, 2016; Sparks et al., 2016; Behzadnia et al., 2018; Vasconcellos et al., 2020)	*Positive* (Valenzuela et al., 2018)	*Positive* (3/3 SS) (Guay et al., 2010a)
Identified		*Positive* (Guay et al., 2013; Sparks et al., 2016; Behzadnia et al., 2018; Vasconcellos et al., 2020) *NS* (Guay et al., 2016)	*NS* (Valenzuela et al., 2018)	*Positive* (3/3 SS) (Guay et al., 2010a)
Introjected		*Positive* (Guay et al., 2013; Sparks et al., 2016) *NS* (Guay et al., 2016; Behzadnia et al., 2018; Vasconcellos et al., 2020)	*NS* (Valenzuela et al., 2018)	
External		*Positive* (Guay et al., 2013) *Negative* (Vasconcellos et al., 2020) *NS* (Guay et al., 2016; Sparks et al., 2016; Behzadnia et al., 2018)	*Positive* (Valenzuela et al., 2018)	

NS = no significant link. SS = school subject.

**Table 2:** Synthesis of the links observed in the studies between motivation and its consequences according to the hierarchical level considered and the operationalization of the motivation used.

	Consequences					
	Achievement		Positive emotions		Negative emotions	
	Contextual	Situational	Contextual	Situational	Contextual	Situational
RAI	*Positive* (Grolnick et al., 1991; Fortier et al., 1995; Guay and Vallerand, 1997; Ratelle et al., 2005)	*Positive* (Black and Deci, 2000)	*Positive* (Miserandino, 1996; Levesque et al., 2004)	*Positive* (Black and Deci, 2000)		*Negative* (Black and Deci, 2000)
AM and CM						
AM	*Positive* (Brunet et al., 2015; Litalien et al., 2015)	*Positive* (De Naeghel et al., 2012; Jeno et al., 2018; Botnaru et al., 2021)	*Positive* (Brunet et al., 2015; Litalien et al., 2015)		*Negative* (Brunet et al., 2015; Litalien et al., 2015)	
CM	*Positive* (Brunet et al., 2015; Litalien et al., 2015) *NS* (Kusurkar et al., 2013)	*Negative* (De Naeghel et al., 2012; Botnaru et al., 2021) *NS* (Jeno et al., 2018)	*Negative* (Brunet et al., 2015; Litalien et al., 2015)		*Positive* (Brunet et al., 2015; Litalien et al., 2015)	
Regulations separately						
Intrinsic	*Positive* (Taylor et al., 2014; Litalien et al., 2015; Orsini et al., 2019; Howard et al., 2021, study 1)	*Positive* (Noels et al., 2001; Leroy and Bressoux, 2016; Lohbeck, 2018) *NS* (Noels et al., 1999; McEown et al., 2014)	*Positive* (Litalien et al., 2015; Howard et al., 2021)		*Negative* (Litalien et al., 2015)	*Negative* (Noels et al., 1999)
Identified	*Positive* (Taylor et al., 2014; Litalien et al., 2015; Orsini et al., 2019; Howard et al., 2021, study 1) *NS* (Cokley et al., 2001; Fairchild et al., 2005)	*NS* (Noels et al., 1999, 2001; McEown et al., 2014; Leroy and Bressoux, 2016; Lohbeck, 2018)	*Positive* (Litalien et al., 2015; Howard et al., 2021)		*Negative* (Litalien et al., 2015)	*Negative* (Noels et al., 1999)
Introjected	*Negative* (Taylor et al., 2014; Litalien et al., 2015, study 1) *NS* (Cokley et al., 2001; Fairchild et al., 2005; Orsini et al., 2019; Howard et al., 2021)	*NS* (Noels et al., 1999, 2001; McEown et al., 2014; Leroy and Bressoux, 2016; Lohbeck, 2018)	*Negative* (Litalien et al., 2015; Howard et al., 2021) *Positive* (Assor et al., 2009, study1; Vallerand et al., 1989; Bailey and Phillips, 2016; Howard et al., 2021)		*Positive* (Litalien et al., 2015)	*NS* (Noels et al., 1999)
External	*Negative* (Taylor et al., 2014; Litalien et al., 2015, study 1) *Positive* (Taylor et al., 2014, study 4) *NS* (Cokley et al., 2001; Fairchild et al., 2005; Orsini et al., 2019; Howard et al., 2021)	*Negative* (Leroy and Bressoux, 2016; Lohbeck, 2018) *NS* (Noels et al., 1999, 2001; McEown et al., 2014)	*Negative* (Litalien et al., 2015; Howard et al., 2021)		*Positive* (Litalien et al., 2015)	*NS* (Noels et al., 1999)

NS = no significant link.

### The school-subject-specificity hypothesis

The school-subject-specificity hypothesis has been developed to explain a non-expected differentiation effect found between school subjects in SDT motivation types. Guay et al. (2010a) investigated variations in motivation across different school subjects (i.e., between school subject differentiation) and demonstrated that the correlations between autonomous motivation for different school subjects were lower than the correlations between controlled motivation for the same school subjects. This differentiated pattern between motivations was not expected nor theoretically conceptualized. Therefore, according to HMIEM, Chanal and Guay (2015) examined primary and secondary students’ autonomous and controlled types considering simultaneously two hierarchical levels of the model: The situational level (i.e., motivations for different school subjects) and the contextual level (i.e., motivations toward school in general). These authors investigated the possibility that the differentiation effect found by Guay et al. (2010a) could be related to the degree of specificity of the motivation types with the situational level in which they are measured. The school-subject-specificity-hypothesis states that AM types are more differentiated between school subjects than CM types because AM types are more specific to the situational level. Indeed, AM types would be more school-subject-specific and therefore more differentiated because their regulatory processes are more specific to the characteristics of the activity. The school offers different activities to children, and they discover early those, which give them pleasure and those which give them less (i.e., intrinsic motivation) but also those to which they will more or less identify themselves (i.e., identified regulation). CM types would be less differentiated and therefore less specific because its internal regulatory processes (i.e., introjected regulation) or external regulatory processes (i.e., external regulation) would not be school-subject-specific but could be present in all school subjects. To test their hypothesis, Chanal and Guay (2015) build structural equation models (i.e., correlated trait-correlated method minus one model; CTCM-1) which permitted to distinguish shared variance attributable to the contextual level (i.e., school) and to the situational level (i.e., school subjects). Their results confirmed the school-subject-specificity hypothesis. Indeed, shared variance at the situational level for AM items was higher than for CM items, demonstrating that AM types are more specific than CM types. The school-subject-specificity hypothesis has recently been replicated and extended to more motivation types of the self-determination continuum (Chanal and Paumier, 2020). Chanal and Paumier (2020), using appropriate statistical models that distinguish between contextual and item level variance, demonstrated also that shared variance for CM items were found to be more related to the item level. More importantly, the relations between motivation types and different constructs at the situational level were found to be dependent on the level of specificity of the motivation considered (Chanal and Guay, 2015; Chanal and Paumier, 2020). Indeed, as presented in the previous sections, AM types were more related to self-concept (Chanal and Guay, 2015) and achievement (Chanal and Guay, 2015; Chanal and Paumier, 2020) than CM types.

### The present study

In the academic context, the motivational sequence (i.e., “antecedents – motivation – consequences”) has mainly been tested either at the contextual level (i.e., academic, or university) or at a situational level (i.e., school subject, or university course). However, research on the school-subject-specificity hypothesis (Chanal and Guay, 2015; Chanal and Paumier, 2020) has shown that the difference in the level of specificity of AM and CM types influenced the existing links with different constructs (i.e., achievement, self-concept). In particular, these studies showed that the most specific motivation types were more strongly associated with constructs than the less specific ones. As a result, the existence of the motivational sequence could depend on the specificity of the motivation type. Considering the specificity hypothesis, this study’s main objective was therefore to evaluate the complete motivational sequence (i.e., “antecedents – motivation – consequences”) described by the HMIEM (Vallerand, 1997) at the situational level controlling for the shared variance of the measures with the contextual level. More specifically, we investigated the impact of the level of specificity of the AM and CM types in different university courses on the relations between antecedents and consequences and student’s motivation. Indeed, the HMIEM model had never considered that motivation types may differ according to their level of specificity. As previously discussed, studies concerning the antecedents and consequences of motivation showed inconsistent results according to the operationalization of motivation used and the hierarchical level considered. The difference in situational specificity level of AM and CM types could explain these inconsistencies. We therefore measured two types of situational antecedents (i.e., self-concept and motivational climate), and two types of situational consequences (i.e., achievement and academic emotions). Our first objective was then to evaluate the repartition of shared variance of motivation types at situational level across multiple sources of variance (i.e., situational, contextual and item levels). More precisely, we postulated that the distribution of the shared variance across these sources would be different for AM and CM types, in confirmation of the school-subject-specificity hypothesis. Following this, our second objective was to evaluate how these differences in distribution of shared variance impact the motivational sequence depicted in HMIEM model at the situational level (i.e., “antecedents – motivation – consequences”). We expected that this motivational sequence at the situational level (i.e., “antecedents – motivation – consequences”) would be demonstrated for AM types but not, or less evidently, for CM types.

Our hypotheses were as follows:

*Hypothesis 1*: According to the school-subject-specificity hypothesis (Chanal and Guay, 2015), we expected that AM would be more specific to the situational level than CM. More specifically, we hypothesized that the quantity of shared variance for items measured at the situational level would be higher for AM than for CM. More specifically, we hypothesized that specificity (i.e., shared variance at the situational level) would be gradually decreasing along the self-determination continuum. In contrast, based on the recent work of Chanal and Paumier (2020), we expected that the quantity of shared variance at the item level would be higher for CM than for AM.

*Hypothesis 2*: We postulated that antecedents (i.e., autonomy-supportive climate, and self-concept) at the situational level would be significantly correlated with AM types but not or less with CM types.

*Hypothesis 3*: We expected that consequences (i.e., academic emotions, and grades) at the situational level would be significantly correlated with AM types but not or less with CM types.

## Materials and methods

### Participants and procedure

Participants were university students in first year of psychology at University of Geneva, Switzerland. Participants completed online questionnaires using Qualtrics^2^ three times during autumn semester: At the beginning (T1: 18–22 October 2017), at the middle (T2: 6–12 November 2017), and the end of the semester (T3: 4–10 December 2017). At time 1, 314 students participated (17.83% male, *M*_age_ = 21.71 years, *SD*_age_ = 4.7 years), 299 at time 2, and 288 at time 3. We address the issue of missing data in the statistical analyses section.

Written consent was required from the participants in order to participate in the study. The ethics commission of the faculty of psychology of the University of Geneva approved this study. The data was obtained and analyzed anonymously. Participants received course credit for their participation.

### Measures

#### Academic motivation

Student’s motivation was measured using a scale recently developed and validated by Sheldon et al. (2017). We assessed five subscales measuring five self-determined motivation types, with four items per subscale. The subscales are as follows: Intrinsic motivation (e.g., “because I enjoy …”), identified regulation (e.g., “because I strongly value …”), positive introjected regulation (e.g., “because I want to feel proud of myself”), negative introjected regulation (e.g., “because I would feel guilty if I did not do …”), external regulation (e.g., “because important people (i.e., parents, professors will like me better if I do …”). In their study, carried out on 4 samples, Sheldon et al. (2017) reported Cronbach’ alphas between 0.80 and 0.94 for intrinsic motivation subscale, between 0.73 and 0.86 for identified regulation subscale, between 0.68 and 0.82 for positive introjected regulation subscale, between 0.77 and 0.86 for negative introjected regulation subscale, and between 0.61 and 0.88 for external regulation subscale. The scale was adapted to assess student’s regulation types at the contextual level (i.e., motivation for studying psychology) and at the situational level (i.e., motivation for five university courses: Statistics, social psychology, cognitive development, psychology of motivation, clinical psychology). These courses were chosen because they are mandatory courses in the first year of psychology studies and therefore taken by all students during the first semester. The same four items were used to assess each motivation type at the contextual and situational levels. The students were asked how much they agreed with each reason “to study psychology” or “to participate in a particular course” on a 7-point Likert scale from 1 (*never*) to 7 (*all the time*). The scale was administered to students at time 2. In our study, the Cronbach’ alphas for each subscale were as follows: αs between 0.92 and 0.96 for intrinsic motivation), between 0.81 and 0.86 for identified regulation, between 0.78 and 0.90 for positive introjected regulation, between 0.73 and 0.84 for negative introjected regulation, between 0.48 and 0.69 for external regulation.

### Autonomy-supportive climate

Autonomy-supportive climate was measured using the Learning Climate Questionnaire (LCQ; Williams and Deci, 1996). The LCQ measures students’ perceptions of autonomy-supportive behaviors of their teachers and contains 15 items (e.g., I feel that my professor provides me choices and options). For each of 15 items, students rate their agreement on a 7-point Likert scale from 1 (*strongly disagree*) to 7 (*strongly agree*). In their study (Williams and Deci, 1996), the Cronbach’ alpha for this scale was 0.96. The scale was adapted to assess the autonomy-supportive climate in two university courses: In statistics and in social psychology. The scale was administered at time 1. Cronbach’s alphas were 0.88 (teacher’s autonomy support in statistics) and 0.90 (teacher’s autonomy support in social psychology).

#### Students’ self-concept

Six items of the Self-Description Questionnaire (Guérin et al., 2003) were used to assess students’ self-concept. In their study, Guérin et al. (2003) reported Cronbach’ alphas between 0.85 and 0.95. The scale was adapted to measure self-concept at the contextual level (i.e., self-concept in psychology studies) and at the situational level (i.e., self-concepts in statistics, social psychology, cognitive development, and psychology of motivation). For each of six items (e.g., “I am doing well in …”), students were asked to rate how much they agreed with each item on a 7-point Likert scale from 1 (*strongly disagree*) to 7 (*strongly agree*). The same six items were used to assess each self-concept at the contextual and situational levels. The scale was administered at time 1. In our study, Cronbach’s alphas for this measure were 0.85 for psychology, 0.91 for statistics, 0.88 for social psychology, 0.85 for cognitive development, and 0.88 for psychology of motivation.

#### Academic emotions

Students’ academic emotions were measured using the Academic Emotions Scale (Govaerts and Grégoire, 2008). The scale assesses seven academic emotions: Enjoyment (4 items, e.g., “I feel great when I study for …”), hope (4 items, e.g., “I feel optimistic about the preparation of the course”), pride (3 items, e.g., “I am proud of the way I am preparing the course”), anxiety (5 items, e.g., “I feel anxious when I study for …”), boredom (3 items, e.g., “I am bored studying for …”), anger (3 items, e.g., “the subjects I have to study irritate me”), shame (4 items, e.g., “I feel ashamed thinking I might have not prepared the course properly”). For each item, students rate their agreement on a 7-point Likert scale from 1 (*strongly disagree*) to 7 (*strongly agree*). In their study, Govaerts and Grégoire (2008) reported the following Cronbach’s alphas: 0.91 for the anxiety subscale, 0.88 for the frustration subscale, 0.87 for the enjoyment subscale, 0.85 for the hope subscale, 0.73 for the shame subscale, and 0.65 for the pride subscale. The scale was adapted to assess these seven emotions in the different courses: In statistics, social psychology, and clinical psychology. The same items were used to assess each emotion in these three courses. The scale was administered at time 3. In our study, Cronbach’s alphas for the various academic emotions subscales across school subjects ranged from 0.83 to 0.86 for enjoyment, from 0.92 to 0.93 for hope, from 0.70 to 0.78 for pride, from 0.90 to 0.92 for anxiety, from 0.85 to 89 for boredom, from 0.84 to 0.92 for anger, and from 0.78 to 0.82 for shame.

#### Students’ final grades

Students’ final grades in five courses (statistics, social psychology, cognitive development, psychology of motivation, clinical psychology) were obtained from the university administration at the end of the semester. For statistics, two grades were obtained: The grade for the practical course and for the theoretical course. In Swiss’ educational system, grades range from 1 to 6, where 6 represents the highest grade and 1 the lowest grade.

### Statistical analyses

#### Correlated trait-correlated method minus one model

The Correlated trait-correlated method minus one (CTCM-1) model (Eid et al., 2003) appeared to be the most suitable model to investigate the specificity of the motivation types. CTCM-1 model is used in multitrait-multimethod research to distinguish the variance due to methods and traits. As explained by Chanal and Guay (2015), this modeling procedure “has the advantage of combining and disentangling variances in measures attributable to a global (i.e., contextual) trait or to a state or method (i.e., specific) measure” (p. 7). Moreover, this model allows investigating the hierarchical structure of academic motivation by considering various school subjects or courses.
Applied to our study, CTCM-1 model allows distinguishing the variance in autonomous and controlled motivation attributable to the contextual level (i.e., motivation for studying psychology) and to the situational, specific level (i.e., university courses). More precisely, intrinsic motivation at the contextual level (i.e., motivation for studying psychology) is considered as a single trait, whereas intrinsic motivations in different university courses are considered as correlated methods or courses deviations from this global trait. The latent construct for contextual intrinsic motivation influences the items of contextual intrinsic motivation and the items of the five courses. The latent constructs for intrinsic motivations in each course (i.e., specific factors) influence items of the corresponding course (e.g., latent constructs for statistics influences statistics measures) over and above the latent construct for contextual intrinsic motivation (i.e., global factor). Thus, the specific latent factors for each course represent deviations from the global factor by capturing the common but specific variance in course items that is above the common variance at the contextual level. The items, which assess regulation at the contextual level, are influenced only by the global factor but not by a specific factor, representing the method minus one part of the CTCM-1 model. This missing “method factor” allows the model to be identified and a unique solution to be obtained for all model parameters.
For each motivation type (i.e., intrinsic, identified, positive introjected, negative introjected, external), we realized a CTCM-1 model. We realized a CTCM-1 model also for self-concept because self-concept can be considered as a multidimensional and hierarchical construct (see Brunner et al., 2010). For other constructs (i.e., autonomy-supportive climate, and academic emotions) except students’ final grades, we conducted confirmatory factor analyses (CFA). Factor scores were calculated for each latent factor and were used in the Pearson correlations analyses to investigate the links between motivation and its antecedents and consequences.

#### Correlation analysis

In order to analyze the links between motivation types and its antecedents, or between motivation types and its consequences, we calculated Pearson correlations between the factor scores extracted from the models of each of the constructs. Then, Chi-Square Tests of Independence were performed to determine whether the proportion of significant correlation between motivation types and constructs (i.e., antecedents, and consequences) was different between autonomous motivation types and controlled motivation types.

#### Missing data

##### Missing data for CTCM-1 and CFA models

The statistical models (i.e., CTCM-1 and CFA models) were built on all the data collected at each of the 3 measurement times. The models for autonomy-supportive climate and self-concept were built on the sample of participants who completed the scales at time 1 (*N* = 314). The models for each motivation types were built on the sample of participants who completed the scale at time 2 (*N* = 299). The model for academic emotions was built on the sample of participants who completed the scale at time 3 (*N* = 288). For each model performed, there was less than 1% missing data (i.e., missing cells). To account for missing data, full information maximum likelihood (FIML) was performed using Mplus (version 7).

##### Missing data for correlation analysis

For each correlation analysis, as the number of participants who completed the scales of the different constructs was not the same, we used the pairwise deletion of missing data method. Correlations between antecedents (i.e., autonomy-supportive climate, and self-concept) and motivation types were calculated on 294 participants. The correlations between motivation types and academic emotions were calculated on 282 participants. The correlations between motivation types and grades were calculated on a sample varying between 228 and 266 participants.

#### Estimation and goodness of Fit

All models were tested with maximum likelihood estimation using robust standard errors (MLR estimation). To evaluate the model fit, we used the chi-square values, the comparative fit index (CFI), the Tucker-Lewis index (TLI), the root mean square error of approximation (RMSEA), and the standardized root mean square residual (SRMR). CFI and TLI values closed to or above.90 and.95 are deemed acceptable and excellent fit to the data, respectively, (Bollen and Curran, 2006). For RMSEA, values closed to or below.08 are indicative of an adequate fit (Hu and Bentler, 1999; Marsh et al., 2005). A value of 0.08 (or lower) for the SRMR is considered indicative of a good model fit (Hu and Bentler, 1999).

#### Parallel item

**Figure 1:**
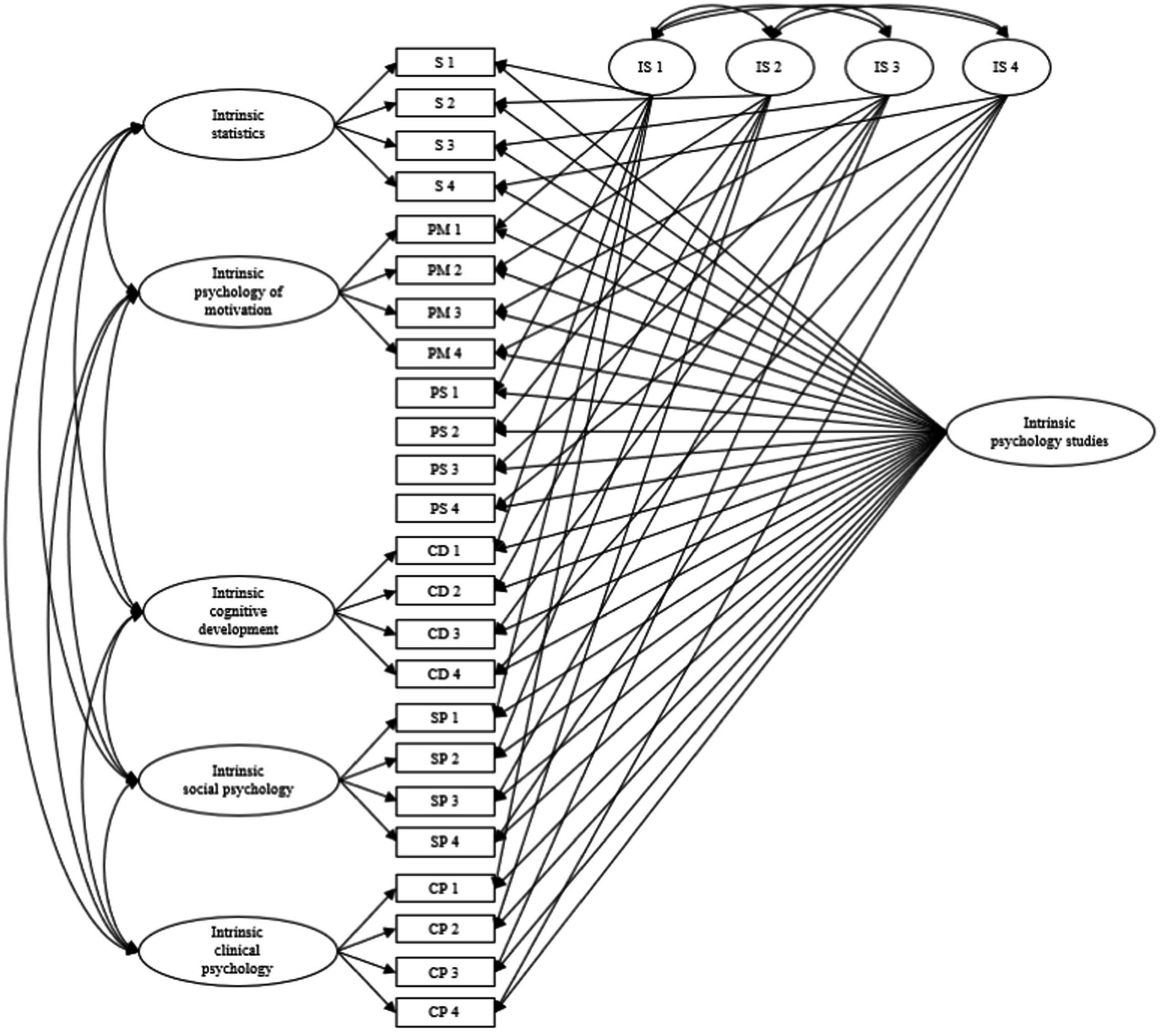
CTCM-1 Model for Intrinsic Motivation. 1–4 = items for statistics; PM 1–4 = items for psychology of motivation; PS 1–4 = items for psychology studies; CD 1–4 = items for cognitive development; SP 1–4 = items for social psychology; CP 1–4 = items for clinical psychology; IS 1–4 = item-specific factors.

Identical items were used to assess the same regulations toward studies and across school subjects. As Chanal and Paumier (2020), we created an item-specific factor for the same item at the situational and contextual levels (see Figure 1 for CTCM-1 model for intrinsic motivation). Thus, for each regulation, the CTCM-1 model allows distinguishing the variance attributable to the contextual, the situational and the items levels.

## Results

Table 3 presents fit indices for each regulation type model. All models show an excellent fit to the data.

**Table 3:** Fit Indices of the models.

Model	Chi^2^	df	*Value of p*	RMSEA	CFI	TLI	SRMR
Intrinsic	271.00	192	0.000	0.04	0.98	0.98	0.03
Identified	277.28	192	0.000	0.04	0.98	0.96	0.03
Positive Introjected	242.05	192	0.008	0.03	0.99	0.98	0.03
Negative Introjected	248.89	192	0.004	0.03	0.99	0.98	0.03
External	378.95	192	0.000	0.06	0.94	0.91	0.04

### The school-subject-specificity hypothesis

Hypothesis 1 was that the more autonomous the motivation types were, the more specific they would be. That is, we expected that the variance of the items shared at the situational level would be greater for the most autonomous motivation types. Table 4 showed the percentage of total variance for each motivation types attributed to the different sources of variance considered (situational, contextual, item, and residual). Results confirmed the school-subject-specificity hypothesis. On average, the percentage of variance shared at the situational level for intrinsic motivation (66% in total variance) and for identified regulation (42% in total variance) were higher than for positive introjected regulation (22% in total variance), negative introjected regulation (26% in total variance) and external regulation (20% in total variance). Moreover, results demonstrated that the percentage of variance shared at the situational level decreased as motivation types become less autonomous except for negative introjected regulation. On average, the percentage of variance shared at the contextual level was higher for intrinsic motivation and identified regulation (14% in total variance) than for positive introjected regulation (1% in total variance), negative introjected regulation (4% in total variance) and external regulation (8% in total variance). More importantly, on average, the percentage of variance shared at the item level was higher for positive introjected regulation (54% in total variance), negative introjected regulation (44% in total variance) and external regulation (38% in total variance) than for intrinsic motivation (4% in total variance) and identified regulation (20% in total variance). These results show that the CM motivation types are more related to the item level than to the contextual level.

**Table 4:** Percentage of the variance due to situational (specific), contextual and item levels on average and for each course by motivation type.

Intrinsic	Specific	Contextual	Item	Residual
Statistics	72	7	4	18
Psychology of motivation	67	16	1	15
Cognitive development	65	18	4	13
Social psychology	59	18	6	17
Clinical psychology	68	13	3	15
Average	66	14	4	16
Identified	Specific	Contextual	Item	Residual
Statistics	45	7	16	32
Psychology of motivation	43	14	20	23
Cognitive development	43	14	23	20
Social psychology	45	13	20	21
Clinical psychology	34	21	19	26
Average	42	14	20	24
Positive introjected	Specific	Contextual	Item	Residual
Statistics	20	2	48	30
Psychology of motivation	23	0	53	24
Cognitive development	13	1	67	19
Social psychology	28	1	48	24
Clinical psychology	26	3	52	20
Average	22	1	54	23
Negative introjected	Specific	Contextual	Item	Residual
Statistics	21	2	53	24
Psychology of motivation	27	4	42	27
Cognitive development	32	4	34	30
Social psychology	25	4	45	25
Clinical psychology	23	4	43	30
Average	26	4	44	27
External	Specific	Contextual	Item	Residual
Statistics	17	2	37	44
Psychology of motivation	23	10	37	29
Cognitive development	21	8	43	27
Social psychology	17	9	42	32
Clinical psychology	23	9	33	35
Average	20	8	38	34

### Motivation and antecedents at the situational level

The correlations between each motivation type and antecedents at the situational level are presented in Table 5 for autonomy-supportive climate and in Table 6 for self-concept. As expected for hypothesis 2, Chi-Square Test of Independence indicated a significant relationship between significance of correlations with antecedents (i.e., autonomy-supportive climate, and self-concept) and motivation types in a corresponding course (i.e., AM vs. CM), *X^2^* (1, *N* = 30) = 20.00, *p* < 0.001. Results indicated a higher proportion of significant correlations between antecedents (i.e., autonomy-supportive climate, and self-concept) and AM types (12 on 12 = 100%) in a corresponding course than between antecedents and CM types (3 on 18 = 16.67%).

**Table 5:** Correlations between autonomy-supportive climate and motivation types in corresponding courses (*n* = 294).

Motivation type	Autonomy-supportive climate	
	Statistics	Social psychology
Intrinsic	**0.25**	**0.32**
Identified	**0.16**	**0.29**
Positive introjected	−0.05	0.09
Negative introjected	0.03	**−0.13**
External	−0.08	−0.10

Significant correlations at *p* < 0.05 or less are shown in bold.

**Table 6:** Correlations between self-concept and motivation types in corresponding courses (*n* = 294).

Motivation type	Self-concept
Intrinsic	
Statistics	**0.46**
Psychology of motivation	**0.42**
Cognitive development	**0.38**
Social psychology	**0.46**
Identified	
Statistics	**0.33**
Psychology of motivation	**0.30**
Cognitive development	**0.34**
Social psychology	**0.35**
Positive introjected	
Statistics	0.02
Psychology of motivation	0.03
Cognitive development	0.10
Social psychology	**0.15**
Negative introjected	
Statistics	−0.11
Psychology of motivation	−0.05
Cognitive development	0.08
Social psychology	**−0.17**
External	
Statistics	−0.04
Psychology of motivation	−0.05
Cognitive development	−0.01
Social psychology	−0.11

Significant correlations at *p* < 0.05 or less are shown in bold.

More precisely, concerning the links between motivation and autonomy-supportive climate, a higher proportion of significant correlations were found for AM (4 on 4 = 100%) than for CM types (1 on 6 = 16.67%). In the statistics course, significant and positive correlations were found between autonomy-supportive climate and intrinsic motivation (*r* = 0.25, *p* < 0.001), and between climate and identified regulation (*r* = 0.16, *p* = 0.007). In contrast, no significant correlations were found between autonomy-supportive climate and controlled motivation types in statistics (i.e., positive introjected, negative introjected and external regulations). In the social psychology course, significant and positive correlations were found between autonomy-supportive climate and intrinsic motivation (*r* = 0.32, *p* < 0.001) and between climate and identified regulation (*r* = 0.29, *p* < 0.001). Only one significant correlation was found for controlled motivation types in social psychology: between climate and negative introjected regulation (*r* = −0.13, *p* = 0.029).

Concerning correlations between self-concept and motivation types, results showed that self-concept was more related to AM (8 on 8 = 100% of significant correlations) than CM types (2 on 12 = 16.67% of significant correlations). Indeed, for intrinsic motivation, all correlations between students’ self-concept and motivation in the corresponding course were significant and positive (*r* = 0.46, *r* = 0.42, *r* = 0.38, and *r* = 46, for statistics, psychology of motivation, cognitive development, and social psychology, respectively, *ps* < 0.001). For identified regulation, all correlations between students’ self-concept and motivation in the corresponding course were also significant and positive (*r* = 0.33, *r* = 0.30, *r* = 0.34, and *r* = 0.35, for statistics, psychology of motivation, cognitive development, and social psychology, respectively, *ps* < 0.001). It is important to note that correlations between self-concept in a corresponding course were higher for intrinsic motivation than for identified regulation. This result is in conformity with the school-subject-specificity hypothesis because intrinsic motivation was found to be more specific than identified regulation. In contrast, for CM types, 16.67% of correlations appeared to be significant. These significant correlations were found between students’ self-concept and positive introjected regulation in social psychology (*r* = 0.15, *p* = 0.009) and between self-concept and negative introjected regulation in social psychology (*r* = −0.17, *p* = 0.003). However, even in line with previous results (Assor et al., 2009) and with theoretical postulates, these correlations also supported the school-subject-specificity hypothesis because these correlations were lower than those between students’ self-concept and AM types.

### Motivation and consequences at the situational level

The correlations between each motivation type and consequences at the situational level are presented in Table 7 for academic emotions and in Table 8 for grades. As expected for hypothesis 3, Chi-Square Test of Independence between correlations’ significance and motivation types was significant (*X^2^* (1, *N* = 135) = 32.20, *p* < 0.001). Results indicated a higher proportion of significant correlations between consequences (i.e., academic emotions, and grades) and AM types (39 on 54 = 72.22%) in a corresponding course than between consequences and CM types (18 on 81 = 22.22%).

**Table 7:** Correlations between motivation types and academic emotions in corresponding courses (*n* = 282).

	Academic emotion						
Motivation type	Enjoyment	Hope	Pride	Boredom	Anger	Anxiety	Shame
Intrinsic							
Statistics	**0.54**	**0.37**	**0.39**	**−0.54**	**−0.53**	**−0.34**	**−0.36**
Social psychology	**0.46**	**0.26**	**0.26**	**−0.51**	**−0.47**	**−0.19**	−0.11
Clinical psychology	**0.30**	**0.12**	**0.22**	**−0.31**	**−0.29**	−0.09	−0.05
Identified							
Statistics	**0.43**	**0.34**	**0.31**	**−0.39**	**−0.42**	**−0.31**	**−0.31**
Social psychology	**0.39**	**0.24**	**0.21**	**−0.45**	**−0.40**	**−0.15**	−0.07
Clinical psychology	**0.19**	0.11	**0.19**	**−0.20**	**−0.17**	−0.05	−0.02
Positive introjected							
Statistics	0.06	0.09	**0.18**	−0.02	−0.02	−0.01	−0.08
Social psychology	**0.16**	**0.15**	0.11	**−0.17**	**−0.16**	−0.01	0.08
Clinical psychology	0.03	0.08	0.06	0.00	0.03	0.07	**0.12**
Negative introjected							
Statistics	−0.11	**−0.13**	−0.06	0.11	**0.14**	**0.18**	**0.13**
Social psychology	**−0.18**	**−0.15**	−0.09	**0.17**	**0.19**	**0.21**	**0.17**
Clinical psychology	0.00	−0.01	−0.04	0.06	0.07	0.11	**0.16**
External							
Statistics	−0.03	0.00	0.02	0.05	0.03	−0.03	0.01
Social psychology	−0.11	0.01	0.00	0.10	0.11	0.06	0.09
Clinical psychology	−0.05	−0.04	−0.03	−0.01	−0.01	0.03	0.07

Significant correlations at *p* < 0.05 or less are shown in **bold**.

**Table 8:** Correlations between motivation types and grades in corresponding courses.

Motivation type	Grade					
	Statistics *T* (*n* = 241)	Statistics *P* (*n* = 257)	Psychology of motivation (*n* = 232)	Cognitive development (*n* = 228)	Social psychology (*n* = 266)	Clinical psychology (*n* = 262)
Intrinsic	**0.31**	**0.24**	0.04	0.09	0.11	0.08
Identified	**0.27**	**0.22**	0.04	0.06	0.09	0.05
Positive introjected	**0.19**	0.09	0.01	−0.07	0.02	−0.01
Negative introjected	−0.10	0.01	0.05	−0.05	0.08	0.08
External	−0.03	0.00	0.05	0.05	0.02	0.01

Significant correlations at *p* < 0.05 or less are shown in **bold**. Statistics *T* = statistics theoretical course; Statistics *P* = statistics practical course.

More precisely, concerning the links between motivation and academic emotions, a more important number of significant correlations were found for AM (35 on 42 = 83.33%) than for CM types (17 on 63 = 26.98%). For intrinsic motivation, 85.71% of correlations between this motivation and emotions in a corresponding course, were significant. More specifically, all correlations between intrinsic motivation and positive emotions (i.e., enjoyment, hope, and pride) in the corresponding course were significant and positive (0.12 < *rs* < 0.54, *ps* < 0.05). Correlations between intrinsic motivation and negative emotions (i.e., boredom, anger, anxiety, and shame) in the corresponding course were significant and negative (−0.54 < *rs* < −0.19, *ps* < 0.01), except for shame in social psychology and for shame and anxiety in clinical psychology. For identified regulation, 80.95% of correlations between this regulation and emotions in the corresponding course were significant. Specifically, correlations between identified regulation and positive emotions (i.e., enjoyment, hope, and pride) in the corresponding course were significant and positive (0.19 < *rs* < 0.43, *ps* < 0.01), except for hope in clinical psychology. Correlations between identified regulation and negative emotions (i.e., boredom, anger, anxiety, and shame) in the corresponding course were significant and negative (−0.45 < *rs* < −0.15, *ps* < 0.05), except for shame in social psychology and for anxiety and shame in clinical psychology. Concerning positive introjected regulation, 28.57% of correlations between this regulation and emotions in the corresponding course, were significant. Specifically, positive introjected regulation was positively correlated with pride in statistics (*r = 0*.18, *p* = 0.002), with enjoyment and hope in social psychology (0.15 < *rs* < 0.16, *ps* < 0.05), and with shame in clinical psychology (*r* = 0.12, *p* = 0.040). In social psychology, negative and significant correlations were found between positive introjected regulation and boredom (*r* = −0.17, *p* = 0.005) and anger (*r* = −0.16, *p* = 0.008). Concerning negative introjected regulation, 52.38% of correlations between this regulation and emotions in the corresponding course were significant. More precisely, negative introjected regulation correlated negatively with hope in statistics (−0.13, *p* = 0.032) and with enjoyment and hope in social psychology (−0.18 < *rs* < −0.15, *ps* < 0.05). In contrast, negative introjected regulation correlated positively with shame in all courses (0.13 < *rs* < 0.17, *ps* < 0.05), with anxiety and anger in statistics and in social psychology (0.14 < *rs* < 0.21, *ps* < 0.05), and with boredom in social psychology (*r* = 0.17, *p* = 0.003). Finally, no significant correlation was found between extrinsic regulation and emotions in the corresponding course. In sum, as predicted, a more important number of significant correlations were found between academic emotions and AM types in a corresponding course (83.33% of significant correlations) than between academic emotions and CM types in a corresponding course (26.98% of significant correlations).

Concerning correlations between motivation types and grades, 33.33% of correlations were significant for AM types, whereas 5.56% were significant for CM types. More precisely, intrinsic motivation and identified regulation in statistics were positively related with grade in statistics theoretical course (*r* = 0.31 and *r* = 0.27, respectively, *ps* < 0.001) and with grade in statistics practical course (*r* =0. 24 and *r* = 0.22, respectively, *ps* < 0.001). Concerning correlations between grades and CM types in a corresponding school subject, only one correlation was found to be significant (5.56%). Indeed, positive introjected regulation in statistics was positively correlated with grade in statistics theoretical course (*r* = 0.19, *p* = 0.004).

## Discussion

The purpose of this study was to investigate the impact of the difference in specificity for autonomous and controlled motivation on the motivational sequence described in the HMIEM model (i.e., “antecedents – motivation – consequences”). More precisely, we expected that this motivational sequence would occur more at the situational level for AM than for CM types, because AM types have been found to be more specific to the situational level than CM types.

### Specificity of autonomous and controlled motivation

As previously demonstrated (Chanal and Guay, 2015; Chanal and Paumier, 2020), our research confirmed that AM types were more specific to the situational level than CM types. In addition, we expected that the specificity of the motivations would decrease gradually as motivation becomes less autonomous. Our assumption was partially confirmed. The specificity decreased as motivation types became less autonomous except for the negative introjected regulation one whose specificity was lower than for the positive introjected one. These results mean that university students in psychology may have different levels of autonomous motivations in the courses they have during their studies. Thus, students might be strongly autonomously motivated by the social psychology course but be weakly autonomously motivated by the statistics course. On the contrary, due to their low specificity, the levels of students’ controlled motivations would tend to be similar across the different psychology courses. For example, students with strong controlled motivations for social psychology course would also tend to have strong controlled motivations for statistics course. Our results also showed that CM types were not more related to the contextual level but more related to the item level, in accordance with Chanal and Paumier (2020). These results highlight the necessity to investigate the motivation toward various situational (i.e., school subjects or university courses) and contextual (i.e., school or academic) situations simultaneously to better understand the part of the assessment that is really concerned in a particular specific school subject or university course and not on other sources of shared variances. More precisely, it seems crucial to assess AM types simultaneously across various courses or school subjects because AM types have been found to be differentiated between these situational situations.

### Antecedents and consequences of autonomous and controlled motivation

#### Antecedents

Because AM types were found to be more specific than CM types, we hypothesized that we would find a more important number of significant links between antecedents and consequences with AM compared to with CM types. Concerning autonomy-supportive climate, results confirmed our hypothesis. Autonomy-supportive climate was positively and significantly associated with AM types in a corresponding course (i.e., in statistics and in social psychology) and no significant relations were found between autonomy-supportive climate and CM types in a corresponding course, except for negative introjected regulation in social psychology. Note that the only significant relation found for CM types was a negative one between climate and negative introjected regulation in social psychology. This relation can be explained according to our hypothesis by the fact that the negative introjected regulation was almost as specific as AM types on average. This negative relation is also in line with SDT predictions (Ryan and Deci, 2017). Taken together, these results suggest that the relations between climate and motivation depend on the situational specificity of the motivation types. Indeed, because AM types are more specific to the situational level, climate introduced by the teacher in a particular situation (e.g., a statistical course) is related to students’ autonomous motivation in this particular course. In contrast, because CM types are less specific to the situational level, climate in a particular situation is not related to the controlled motivation.

Concerning student’s self-concept, as expected, a more important number of significant relations were found between self-concept and AM types at the situational level in comparison to between self-concept and CM types. These results indicated that the more students felt competent in a particular course, the more autonomously motivated they were in this course. Globally, our results are in line with studies considering each motivation type separately and carried out at the situational level, that showed that self-concept was positively associated with AM types (Guay et al., 2010a; Chanal and Guay, 2015) but non significantly with CM types (Chanal and Guay, 2015). Moreover, we have to note that the two significant correlations relating CM types and students’ self-concept were found for the two types of introjected regulations. More specifically, a positive link was found between self-concept and positive introjected regulation and a negative link was found between self-concept and negative introjected in social psychology. These significant links are not surprising because introjected regulation, focusing on the maintenance or enhancement of self-worth (Assor et al., 2009), is related to one’s perceptions of oneself of which the self-concept is a component. However, it is interesting to note that self-concept could play a role in the approach (i.e., positive introjected) or avoidance (i.e., negative introjected) orientations related to introjected regulation. Indeed, the more competent students feel in a course, the more they will participate in this course in order to increase self-esteem and feel proud of themselves (i.e., positive introjected). In contrast, the more competent students feel in a course, the less they will be motivated by guilt and shame in this course (i.e., negative introjected).

#### Consequences

Concerning academic emotions, as predicted, our results indicated that the relations between motivation and academic emotions depended on the specificity of the motivation types. Indeed, a higher number of significant correlations were found between academic emotions and AM types in a corresponding course (i.e., statistics, social psychology, and clinical psychology), than between academic emotions and CM types. Indeed, 35 significant links on 42 were observed for AM types, whereas only 17 significant links on 63 were found for CM types. More precisely, AM types (i.e., intrinsic and identified regulations) were positively associated with positive emotions and negatively associated with negative emotions according to other studies whatever the hierarchical level considered and the operationalization of the motivation used (e.g., Miserandino, 1996; Noels et al., 1999; Black and Deci, 2000; Levesque et al., 2004; Litalien et al., 2015; Howard et al., 2021). Our results highlight the positive between AM types and academic affects by showing that the more the students were autonomously motivated in a particular course, the more they experienced enjoyment, hope and pride in this course, and the less they experienced boredom and anxiety in this course. While AM types are associated with emotions in a corresponding course, CM types are less or not associated with emotions in a corresponding course. This result is in line with a previous study at the situational level, which showed no significant link between CM and affects (Noels et al., 1999), but is contrary to results from other studies at the contextual level that found no significant links between CM and affects (Ryan and Connell, 1989; Litalien et al., 2015; Bailey and Phillips, 2016). These mixed results dependent on the hierarchical level considered can be explained by the specificity of the motivation types at the situational level. When evaluated at the situational level, no relations were found, but when CM and affects are measured at the contextual level, significant relations are found. Some significant correlations between CM and academic emotions are worth mentioning. More specifically, differentiated associations with affects also appeared for the different types of introjected regulation. The more students demonstrated positive introjected regulation, the more they experienced positive emotions (e.g., enjoyment in social psychology) and the less they experienced negative emotions (e.g., anger in social psychology). Conversely, the more students demonstrated a negative introjected regulation, the less they experienced positive emotions (e.g., enjoyment in social psychology) and the more they experienced negative emotions (e.g., anger in social psychology). According to Assor et al. (2009), it seems essential to evaluate these two types of introjected regulation in order to examine more precisely the positive or negative impact of introjected regulation. Moreover, the positive or negative orientations of introjected regulation may explain why in some studies, introjected regulation is positively (e.g., Ryan and Connell, 1989) or negatively (Litalien et al., 2015) associated with positive emotions.

Concerning the links between motivation and grades, our hypothesis is partially confirmed. Only one significant link was found for CM types, whereas very few relations were observed for AM types. The lack of links between CM types and grades in a corresponding course is in line with some studies (Noels et al., 1999, 2001; McEown et al., 2014) and consistent with our hypothesis. For AM types, results showed that only AM types in statistics are statistically and positively associated with grades in statistics theoretical and practical courses, but no significant relations were found for the other courses (i.e., psychology of motivation, cognitive development, social psychology, and clinical psychology).

### Implications

A key point of the HMIEM concerns the motivational sequence between antecedents and consequences of motivation at different hierarchical levels and for all motivation types described by SDT. This theoretical model had never considered that motivational regulations could differ according to their specificity to the situational level and therefore that the motivational sequence could depend on AM and CM types levels of specificity. Our results demonstrated that the motivational sequence at the situational level is demonstrated for AM but not for CM types. Using RAI in studies investigating the HMIEM and focusing on the situational and contextual level sequences only, previous research was not able to disentangle the true relations that exist between antecedents and consequences of motivation types in a particular situation controlling for shared variance of different motivational constructs assessed together. Therefore, the specificity of regulations should be taken into account in future studies that evaluate the motivational sequence at different levels of generality. In addition, the specificity hypothesis brings into question the use of RAI to operationalize motivation. Indeed, the RAI considers that each of the motivation types coming from the same hierarchical level has the same impact since the coefficients used are moderated only by the level of self-determination of the motivation and not by the level in which the motivation is measured. However, the motivation types were found to be non-equally specific to the level at which they are measured. Therefore, we believe that studies that examine the sequence between motivation and its antecedents and consequences should not use the RAI.

Our results showed that the climate introduced by the teacher in a particular course was associated with the students’ autonomous motivation but not with their controlled motivation in this course. This result has an important implication for interventions, which aim to promote autonomy support by teacher. Indeed, because of the specificity of the motivation types, autonomy-supportive interventions in a school subject or in a course could increase intrinsic motivation and identified regulation but could have no impact on controlled motivations (i.e., introjected and external regulations). Our assumption is in line with Guay et al. (2016) results’ which showed that an autonomy-supportive intervention for the teacher in the writing class increased only students’ intrinsic motivation in writing but had no effect on other motivation types.

### Future directions

In future research, antecedents (e.g., climate or basic psychological needs satisfaction) and consequences (e.g., academic emotions) could be assessed at various hierarchical levels (i.e., contextual and situational levels) to better understand the links between motivation and these variables and the links between levels. Indeed, we demonstrated that because AM types were more specific, AM types were strongly related to its antecedents and consequences assessed at the situational level. In contrast, CM types were less specific and, as a result, were weakly associated with its antecedents and consequences at the situational level. Our results also showed that the proportion of variance shared at the item level for CM types were higher than the ones shared at the contextual and situational levels. In line with this, it would be important to investigate other factors (i.e., antecedents) that influence CM types, and which are the consequences of this motivation. This issue could be examined in future research by measuring consequences at the global level (e.g., need satisfaction at global level, autonomy support in life in general, the tree causality orientations described by SDT, personality).

### Limitations

A first limitation is the low values of Cronbach’s alphas obtained for the external regulation subscale (α between 0.48 and 0.69). The results obtained for this regulation should be taken with caution. However, these results are in line with Sheldon’s validation study, which showed that the Cronbach’s alphas, calculated in several samples (i.e., 4 samples), were also lower for external regulation (α between 0.61 and 0.88) than for the other types of motivation (α between.68 and.94). Then, it is possible that the low Cronbach’s alphas values obtained for the external regulation subscale are related to the low specificity of the external regulation, because the variance of the items of the external regulation was weakly attributed to the contextual factor and the situational factors. Second, although we assessed antecedents at time 1, motivation types at time 2 and consequences at time 3, our design being non-experimental, the results do not permit us to infer about causality. Third, the results of this study showed that AM types were more related to its antecedents (i.e., autonomy-supportive climate, and self-concept) and consequences (i.e., grades, and academic emotions) than CM types. However, it is important to generalize these results to other motivational antecedents (e.g., need satisfaction) and consequences (e.g., persistence). Finally, as we measured various concepts toward different courses simultaneously, so that the scale would not be too long to complete by the students, we made the choice, for certain scale (i.e., autonomy-supportive climate, academic emotions, self-concept), to assess them in only few courses but not in all courses.

## Conclusion

This study examined the links between motivation types and their antecedents and consequences in the light of the school-subject-specificity hypothesis. Our results demonstrate that motivation types are not equally specific to the hierarchical level in which they are assessed. Indeed, autonomous motivation is more specific than controlled motivation. More importantly, the specificity of the regulations has an impact on the motivational sequence (i.e., antecedents – motivation – consequences) described by the HMIEM (Vallerand, 1997). This motivational sequence was observed more often at the situational level for AM than for CM types. In fact, AM types were significantly associated with autonomy-supportive climate and emotions in all corresponding courses. On the contrary, CM types were less significantly associated with autonomy-supportive climate and emotions in a corresponding course. These findings have important implications for research by showing that it is essential to examine AM and CM types of students toward various school subjects or courses simultaneously to get an accurate understanding of the motivational mechanisms at work in the academic context.

## Data availability statement

The raw data supporting the conclusions of this article will be made available by the authors, without undue reservation.

## Ethics statement

The studies involving human participants were reviewed and approved by ethics commission of the faculty of psychology of the University of Geneva. The patients/participants provided their written informed consent to participate in this study.

## Author contributions

DP and JC conceived and designed the study, acquired data, and conducted statistical analyses and interpretation. DP wrote the article with the approval of JC, who has critically revised the content. All authors read and approved the final manuscript.

## Funding

Open access funding is provided by the University of Geneva.

## Conflict of interest

The authors declare that the research was conducted in the absence of any commercial or financial relationships that could be construed as a potential conflict of interest.

## Publisher’s note

All claims expressed in this article are solely those of the authors and do not necessarily represent those of their affiliated organizations, or those of the publisher, the editors and the reviewers. Any product that may be evaluated in this article, or claim that may be made by its manufacturer, is not guaranteed or endorsed by the publisher.

## Footnotes

^1^The most common formula for RAI is: 2(intrinsic) + 1(identified)–1(introjection)–2(external) (Grolnick and Ryan, 1989).

^2^ http://qualtrics.com
